# ICTV Virus Taxonomy Profile: *Asfarviridae*

**DOI:** 10.1099/jgv.0.001049

**Published:** 2018-03-22

**Authors:** Covadonga Alonso, Manuel Borca, Linda Dixon, Yolanda Revilla, Fernando Rodriguez, Jose M. Escribano, ICTV Report Consortium

**Affiliations:** ^1^​ Department of Biotechnology, Instituto Nacional de Investigación y Tecnología Agraria y Alimentaria (INIA), Madrid 28040, Spain; ^2^​ Agricultural Research Service, Plum Island Animal Disease Center, Greenport, NY 11944, USA; ^3^​ The Pirbright Institute, Ash Road, Pirbright, Woking, Surrey GU24 0NF, UK; ^4^​ Centro de Biología Molecular Severo Ochoa (CSIC-UAM), Universidad Autónoma de Madrid, Madrid, Spain; ^5^​ Centre de Recerca en Sanitat Animal, IRTA, (CReSA, IRTA-UAB), Bellaterra, Spain

**Keywords:** *Asfarviridae*, ICTV Report, Taxonomy, African swine fever virus

## Abstract

The family *Asfarviridae* includes the single species *African swine fever virus*, isolates of which have linear dsDNA genomes of 170–194 kbp. Virions have an internal core, an internal lipid membrane, an icosahedral capsid and an outer lipid envelope. Infection of domestic pigs and wild boar results in an acute haemorrhagic fever with transmission by contact or ingestion, or by ticks of the genus *Ornithodoros*. Indigenous pigs act as reservoirs in Africa, where infection is endemic, and from where introductions occur periodically to Europe. This is a summary of the International Committee on Taxonomy of Viruses (ICTV) Report on the taxonomy of the *Asfarviridae*, which is available at www.ictv.global/report/asfarviridae.

## Virion

**Fig. 1.**
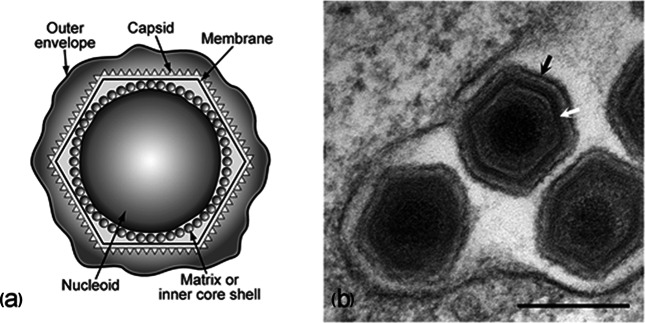
Electron microscope images of virions of African swine fever virus. (a) Diagram of extracellular virions showing nucleoid, inner core shell, internal membrane, capsid and outer envelope. (b) Extracellular virions. The black arrow shows the outer envelope and the white arrow shows the virus membrane. Bar, 200 nm. (Image kindly provided by P. Hawes, Institute for Animal Health, UK).

The virion of African swine fever virus consists of a nucleoprotein core structure, 70–100 nm in diameter, surrounded by an internal lipid layer, an icosahedral capsid and a dispensable external lipid-containing envelope [1]. The capsid exhibits icosahedral symmetry (*T*=189–217) corresponding to 1892–2172 capsomers. The extracellular enveloped virions are 175–215 nm in diameter (Table 1 and Fig. 1).

**Table 1.** Characteristics of the family *Asfarviridae*

Typical member:	African swine fever virus BA71V (U18466), species *African swine fever virus*, genus *Asfivirus*
Virion	Multiple layers of core, internal envelope, capsid and external envelope.
Genome	Linear dsDNA, 170–194 kbp with complementary terminal loops
Replication	Cytoplasmic with an early nuclear phase. Head-to-head concatemer replicative intermediates. Transcription and RNA processing use virus-encoded enzymes. Polyprotein processing by a virus protease yields multiple subunit structural proteins
Translation	From mRNAs with 5′-caps and 3′-polyadenylation
Host range	Domestic pig, wild boar, warthog and bush pig; transmitted by contact and ingestion, and by *Ornithodoros* ticks
Taxonomy	Single species in the single genus *Asfivirus*

## Genome

The genome consists of a single molecule of linear, covalently closed-ended dsDNA of 170–194 kbp. The terminal loops are present as two flip-flop forms that are inverted and complementary. Adjacent to both termini are arrays of directly repeated 2.1 kbp units [2].

## Replication

**Fig. 2.**
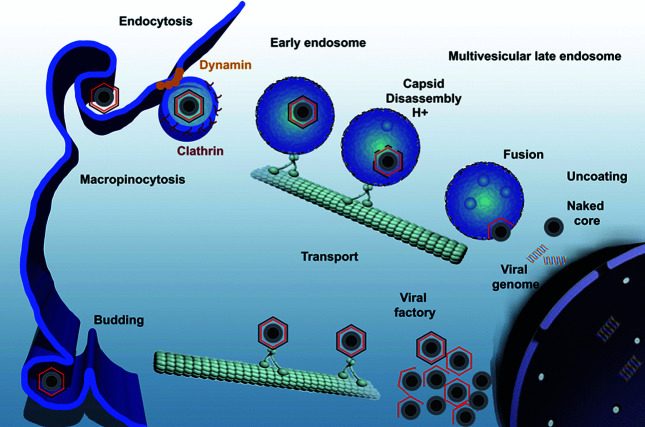
African swine fever virus infectious cycle. Virus enters the cell by clathrin-mediated endocytosis and macropinocytosis. Virions progress through the endocytic pathway and uncoat at late endosomes, where viral decapsidation and then fusion occur. This liberates viral cores to start replication in a cytoplasmic site at the microtubule-organizing centre known as the viral factory. Newly synthesized virions are assembled in the viral factory and exit the cell by budding. Figure reproduced with permission from [8].

African swine fever virus replicates in cell types of the mononuclear–phagocytic system, including fixed-tissue macrophages. Virus enters cells by clathrin-mediated and dynamin-dependent endocytosis and macropinocytosis [3], and requires entry to the endosomal pathway for uncoating (Fig. 2). Viral uncoating relies on host factors during endosomal passage, and capsid disassembly occurs at the acid pH of the endosomal lumen [3]. Cellular lipids are also required in this process. After capsid degradation and membrane fusion, viral cores exit endosomes. The ubiquitin–proteasome system is involved in final degradation of the viral cores to set free the viral DNA in order to start replication [4]. Virus DNA replication and assembly take place in perinuclear factory areas, to which the virus is transported in association with the microtubular motor light-chain dynein. Newly assembled virions exit the viral factories in association with kinesin to be finally released from the cell by budding [5].

## Pathogenicity

African swine fever virus causes a deadly infection of domestic pigs and wild boar, with clinical symptoms of haemorrhagic fever, but it can also cause subacute and chronic disease. African warthogs and bush pigs act as virus reservoirs with persistent, inapparent infections. In the 1960s the virus spread from West Africa to Europe and South America, where it remained endemic for 30 years until it was eradicated everywhere except Sardinia. More recently, virus has spread from Africa to Europe through the Caucasus.

Immunity acquired by non-lethal infection or vaccination is associated with the presence of neutralising antibodies [6].

## Taxonomy

The species *African swine fever virus* was once included in the family *Iridoviridae*, but is now assigned to the genus *Asfivirus*, family *Asfarviridae*. The unclassified faustoviruses, kaumoebavirus and Pacmanvirus [7], have about 30 genes related to those of African swine fever virus, but their genomes are considerably larger (about 400 kbp compared to 170–194 kbp).

## Resources

Full ICTV Online (10th) Report: www.ictv.global/report/asfarviridae.
